# Supplementary material for: Characterization of repeat arrays in ultra‐long nanopore reads reveals frequent origin of satellite DNA from retrotransposon‐derived tandem repeats
Source: Plant J. 2019 Nov 3;101(2):484–500. doi: 10.1111/tpj.14546 (PMC7004042; doi:10.1111/tpj.14546)
Supplement: Supplementary file 2 — Table S1. Similarity hits of L. sativus satellite repeats to the repeat clustering data from two related Lathyrus species. [file TPJ-101-484-s002.pdf]

Supplementary Tab. 1. Similarity hits of *L. sativus* satellite repeats to the repeat clustering data (Macas et al., 2015) from two related *Lathyrus* species

	L. vernus				***L. latifolius***			
**Satellite repeat**	Hit score [(a)]	Cluster[(b)]	Annotation [(b)]	tandem subrepeats [(c)]	Hit score [(a)]	Cluster [(b)]	Annotation [(b)]	tandem subrepeats [(c)]
**FabTR-54**	3e-05, 24/24 (100%)	CL87	Putative LTR-retrotransposon	Yes	1e-06, 26/26 (100%)	CL135	Dispersed repeat	Yes
**FabTR-55**	3e-14, 92/113 (81%)	CL87	Putative LTR-retrotransposon	Yes	3e-64, 145/152 (95%)	CL150	Dispersed repeat	Yes
**FabTR-57**	2e-33, 99/107 (92%)	CL82	LTR/gypsy/ Ogre	Yes	1e-54, 120/123 (97%)	CL5	Putative LTR-retrotransp.	Yes

[(a)] BLASTn hit score is provided as E-value, number of identities/hit length (% similarity)
[(b)] Cluster numbers and their annotations correspond to the repeat analysis described in Macas et al. (2015)
[(c)] Presence of short, tandem subrepeats in contigs assembled from the repeat clusters
