# Supplementary material for: Characterization of repeat arrays in ultra‐long nanopore reads reveals frequent origin of satellite DNA from retrotransposon‐derived tandem repeats
Source: Plant J. 2019 Nov 3;101(2):484–500. doi: 10.1111/tpj.14546 (PMC7004042; doi:10.1111/tpj.14546)
Supplement: Supplementary file 5 [file TPJ-101-484-s005.docx]

**Supplementary Fig. S1**. Dot-plot sequence similarity comparison of consensus monomer sequences. The sequences are separated by green lines and their similarities exceeding 40% over a 100 bp sliding window are displayed as black dots or diagonal lines.

**Supplementary Fig. S2**. Length distributions of nanopore reads displayed as weighted histograms with bin size of 5 kb, with the last bin including all reads longer than 120 kb. (**A**) Length distributions of raw reads from two sequencing runs and the final set of quality-filtered and size-selected (>30kb) reads used for analysis. (**B**) Length distributions of nanopore reads containing rDNA and satellite repeats.

**Supplementary Fig. S3**. Length distributions of satellite repeat arrays displayed as histograms with bin size of 5 kb, with the last bin including all arrays longer than 120 kb. Arrays which were completely embedded within the reads (red bars) are distinguished from those truncated due to their positions at the ends of the reads (blue bars). Tandem repeats forming long arrays are shown in panel **A**, while the remaining repeats forming predominantly short arrays are in panel **B**.

**Supplementary Fig. S4. (A-D)** Self-similarity dot-plot visualization of FabTR-2 arrays. Tandem repeats are revealed as diagonal lines with spacing corresponding to monomer length. (**A**) Example of a 163 kb read completely made of FabTR-2 array (the periodicity pattern is obscured by the high density of lines). (**B**) Magnification of the 10 kb region highlighted by a red square on panel A. This array is homogenized as ~1300 bp HOR. (**C,D**) Examples of other FabTR-2 periodicities detected in different reads (only 10 kb regions were used for dot-plots to make periodicity patterns comparable with other plots).

**(E-H)** Self-similarity dot-plot visualization of FabTR-53 arrays. (**E**) Example of a 202 kb read completely made of FabTR-2 array (the periodicity pattern is obscured by the high density of lines). (**F**) Magnification of the 10 kb region highlighted by a red square on panel A. (**G,H**) Examples of other FabTR-53 periodicities detected in different reads (only 10 kb regions were used for dot-plots to make periodicity patterns comparable with other plots).

**(I-K)** Dot-plots demonstrating length distribution of FabTR-52 arrays, ranging from short arrays (red circle) embedded within LTR-retrotransposon sequences (**I**) and partially expanded arrays (**J**) to the arrays >100 kb in length which are interrupted by insertions of LTR-retrotransposons (blue circles) (**K**).

**(L-N)** (**L**) Example of LTR-retrotransposon carrying short FabTR-54 and FabTR-56 arrays. Reads with those tandem repeats expanded to long arrays are shown on panels **M** (FabTR-54) and **N** (FabTR-56). The expanded tandem arrays appear as black squares on the dot-plots due to high density of lines.

**(O-Q)** Three types of genome organization of FabTR-58 repeats: (O) short array (marked by red circle) within LTR-retrotransposon, (P) expanded array, (Q) short arrays embedded within a longer tandem repeat monomer.

**Supplementary Fig. S5.** **Detailed periodicity analysis of FabTR-2 and FabTR-53 arrays.** Periodicity analysis using fast Fourier transform (FFT) and autocorrelation function (ACF) are shown as averages of spectra calculated on individual satellite arrays longer than 30 kb. Periodicity spectra from individual arrays are shown as heatmaps with rows corresponding to individual arrays. Autocorrelations are shown separately for individual nucleotides. The array average graphs of FabTR-53 were calculated with all subfamilies combined and the FFT peaks corresponding to different monomer lengths of the three subfamilies are indicated with asterisks.

**Supplementary Fig. S6**. **Distribution of the satellite repeats on the metaphase chromosomes of *L. sativus* (2n = 14)**. The satellites were visualized using FISH, with individual probes labeled as indicated by the color-coded descriptions. The chromosomes counterstained with DAPI are shown in gray.

**Supplementary Tab. 1.** Similarity hits of *L. sativus* satellite repeats to the repeat clustering data (Macas et al., 2015) from two related *Lathyrus* species.
